# Body Dissatisfaction among Young Girls in Recreational Rhythmic Gymnastics

**DOI:** 10.3390/children11060696

**Published:** 2024-06-06

**Authors:** Belén Portas Nuñez, Miguel Adriano Sanchez-Lastra, José C. Diz, Carlos Ayán Pérez

**Affiliations:** 1 University of Vigo, 36310 Vigo, Spain; beportas@alumnos.uvigo.es; 2 Department of Special Didactics, Faculty of Educational Sciences and Sports, University of Vigo, 36310 Vigo, Spainjcdiz@uvigo.es (J.C.D.); 3 Well-Move Research Group, Galicia-Sur Health Research Institute (IISGS), SERGAS-UVIGO, 36312 Vigo, Spain

**Keywords:** body image, mental health, children, sports

## Abstract

Body dissatisfaction is commonly associated with rhythmic gymnastics (RG) practice, but limited research exists on the prevalence of this issue among recreational level practitioners. This study examines body image dissatisfaction among young girls practicing RG recreationally. A total of 88 girls between six and eleven years of age, who participate in RG as an extracurricular activity, were measured and completed the Stunkard pictogram. To create a control group, 88 girls who did not practice RG were also recruited and matched to the gymnasts by age. Results revealed that the mean body mass index values in both groups were within the normal weight range. The mean score for body dissatisfaction was similar between the two groups, with slightly positive values (RG = 0.94; CG = 1.06). The Mann–Whitney U test showed that there was no significant difference in the ratings of actual body size, ideal body size, and body dissatisfaction between the RG and control groups. These findings suggest that practicing RG at a young age is not associated with body dissatisfaction among girls.

## 1. Introduction

The representation of one’s own appearance, including a perceptual body schema, along with the emotions, thoughts, and behaviors associated with it is known as body image [1]. When body dimensions are not accurately estimated, we say that this perception is distorted. Thus, body distortion is reflected in the discrepancy between the body perceived by the person and the actual body [2].

Body image dissatisfaction, a negative attitude from perceived discrepancies between actual and ideal body images [3], is increasing among preadolescent girls, often manifesting as a desire for thinness [4]. However, several authors assert that the beauty stereotypes related to thinness are internalized from as early as early childhood [5]. In the study by Lowes and Tiggemann [6], it was found that the desire to achieve a slimmer figure begins at the age of six. Similarly, Jellinek et al. [7] suggest that these behavioral patterns start to manifest in early childhood, particularly in girls between the ages of six and eight. Other authors, such as Pallan et al. [8] and Tremblay et al. [9], demonstrated that the ideal of thinness is present at even earlier ages, specifically in preschool children. Given the possibility of its presence, this fact deserves further investigation in early ages. This concern is linked to depression [10], eating disorders [11], and poor nutritional status [12], necessitating strategies to mitigate its impact. 

Indeed, the overvaluation of physical appearance and the excessive concern about gaining weight constitute the core of the psychology of eating disorders (EDs). With this in mind, addressing body dissatisfaction is essential in the treatment of these disorders, as well as in preventing relapses [13].

Physical activity, known to inversely correlate with body dissatisfaction and positively with body image, could be an effective strategy [14,15]. One way to increase girls’ physical activity levels is through sporting activities, and rhythmic gymnastics (RG) may be a suitable option as it is popular among this population [16]. Additionally, RG is a practice in schools that is well regarded as an extracurricular activity, as it requires less demanding conditions for its development [17]. Extracurricular activities are presented as an excellent opportunity to promote and encourage physical activity. Moreover, they are especially sought after by the school population [18].

Rhythmic gymnastics has traditionally been stereotyped as a sport for females, as it emphasizes aesthetic appearance and femininity [19]. However, due to the nature of RG, gymnasts are often concerned about their body image, as aesthetic abilities and physical appearance are important factors in determining competition success [20]. Furthermore, research has shown that rhythmic gymnasts are more likely to be dissatisfied with their body image compared to other athletes [21]. However, most research in this area has been conducted with adolescent gymnasts who compete at a high level [22]. For example, Vernetta et al. [23] found that young elite-level rhythmic gymnasts were slightly more dissatisfied with their bodies compared to young elite-level acrobatic gymnasts. Similarly, the results of De bruin et al. [24] indicate the same, revealing that elite gymnasts have a more negative relationship with their bodies compared to lower-level gymnasts.

Little is known about the effects of recreational RG practice on body dissatisfaction in girls. Thus, the idea of increasing physical activity levels among this population through RG practice to improve body image perception remains open for discussion. The aim of this study was to investigate the existence of body image dissatisfaction in a sample of girls who practice RG at a recreational level.

## 2. Materials and Methods

### 2.1. Study Design

This was a cross-sectional study in which data were gathered through anthropometric measurements and the administration of a pictogram.

### 2.2. Participants

The girls who participated in this study were recruited from several clubs located in Galicia (north of Spain) offering RG as an extracurricular activity. To ensure homogeneity, we selected girls aged 6–11 who practiced RG recreationally and excluded those competing at national or international levels. A control group (CG) of six to eleven-year-old girls who did not practice RG, and attended different schools in the regional area, was also recruited and matched 1:1 with gymnasts by age. The first author of this study, a former RG practitioner, personally contacted all the clubs through friends and ex-gymnasts who have become RG coaches. Similarly, she reached out to local schools through teachers she knew who work at those institutions. Informed written consent was obtained from the participants and their parents before participating in the study. The study design was approved by the Ethics Committee of the Faculty of Education and Sports Science (University of Vigo) with code 04-280722.

### 2.3. Measurements

Anthropometrics: We assessed body composition using height (cm) and weight (kg) measurements to calculate BMI (kg/m^2^). All measurements were performed using a digital scale and stadiometer.

The participants were classified according to the World Health Organization’s age-specific distributions of BMI into underweight, normal weight, overweight, and obese.

Body dissatisfaction: We used the Stunkard Pictogram adapted for young people, which consists of seven female silhouette figures that increase gradually in size from very thin (a value of 1) to very obese (a value of 7) [25]. The participants were asked to select the figure that most closely resembled their current body shape, their ideal body shape, and the figure they would like to be seen as by others. A body dissatisfaction score was calculated by subtracting the figure chosen for their current body shape from the figure chosen for their ideal body shape. A positive score indicated dissatisfaction with being smaller, a negative score indicated dissatisfaction with being larger, and a score of zero indicated body satisfaction and no desire for change [26]. The test–retest reliability of the pictogram when administered to children has been found to be acceptable [27]. All assessments were conducted individually during a single RG session held during the last month of the extracurricular activities season by a well-trained ex-gymnast with a degree in Primary Education.

### 2.4. Statistical Analysis

Data are expressed as mean ± SD or as n (%) as appropriate. The internal consistency of the data obtained through the Stunkard Pictogram was analyzed with Cronbach’s alpha, and interpreted as follows: excellent (α < 0.9), moderate (α = 0.8–0.7) or low (α < 0.7) [28]. We compared continuous variables with Mann–Whitney U test or Kruskal–Wallis test if they did not have normal distribution, assessed by the Kolmogorov–Smirnov test. Qualitative variables were analyzed using a Chi-Squared Test. We calculated Spearman’s rank correlation coefficient between BMI and the results of the Stunkard pictogram. All analyses were performed using “SPSS Statistical Software version 19.0” and a two-sided *p* value of less than 0.05 indicated statistical significance.

## 3. Results

We included 176 girls in the study, with both groups (88 RG and 88 CG) having mean BMI values within the normal range. Data derived from the Stunkard Pictogram yielded a moderate internal consistency (α = 0.73). The results revealed that 75% and 62.5% of the girls in the RG and control groups (CG) were of normal weight, respectively (Table 1).

There was no significant difference in body dissatisfaction scores between the groups (RG = 0.94; CG = 1.06; *p*= 0.540).

A Mann–Whitney U test revealed that the girls in the RG group did not significantly differ from those in the CG in their ratings of actual body size, ideal body size, or body dissatisfaction.

Approximately 64% and 67% of the girls in the RG and CG groups chose an ideal figure that was smaller than the one they selected to represent their actual size, respectively (Table 2).

Around 29% of the gymnasts and approximately 23% of the controls chose the same figure to represent their actual and ideal selves. Correlation analysis indicated that both the girls in the RG and CG groups perceived their body image accurately (ρ = 0.587; *p* < 0.001). There was also a positive correlation between BMI and body dissatisfaction (ρ = 0.354; *p* < 0.001). About 86% and 91% of the obese and overweight girls wished to be thinner, respectively (Table 3).

**Figure 1:**
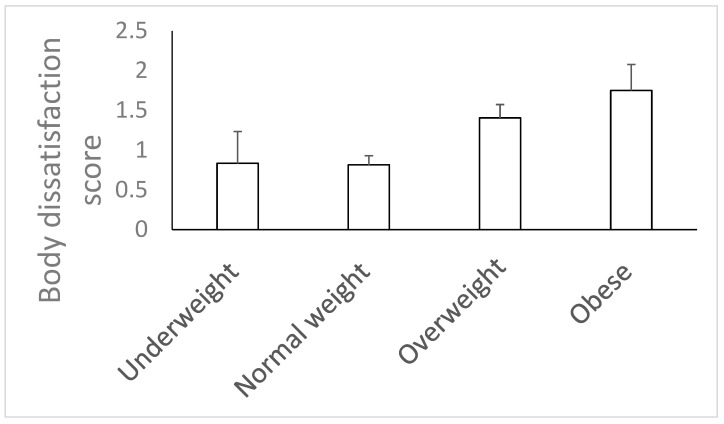
Body dissatisfaction score according to body mass index.

These girls also had the highest mean body dissatisfaction scores (Figure 1). No significant differences were observed between RG and CG girls in any of the variables assessed.

## 4. Discussion

Body image dissatisfaction among children and pre-adolescents has been an understudied topic in comparison to young and adult populations [29]. This study’s findings on body image dissatisfaction among recreational RG practitioners contribute to the limited research focusing on children and preadolescents.

Our results indicate that body dissatisfaction is present among young girls, which echoes previous studies. For example, [27] observed that 55% of 8 to 10-year-old girls (n = 109) were dissatisfied with their body image, with 45 girls wanting to be thinner and 15 wanting to be heavier. Similarly, [30] found that 55% and 19% of 8-to-12-year-old girls (n = 139) preferred to be thinner and bigger, respectively. In the study by [31] conducted with girls aged 5–12 (n = 309), these percentages were 41% and 36%, respectively. These findings suggest that young girls are often dissatisfied with their body image, as previously stated [26]. In our study, less than a third of the girls chose the same figure to represent their actual and ideal self. However, it should be noted that the mean score obtained in the pictogram by our sample when comparing perceived and ideal body image was slightly positive, suggesting that the existing degree of body dissatisfaction was of low magnitude.

Our study found that practicing RG at a recreational level does not influence body dissatisfaction in young girls. This lack of influence may be attributable to the non-competitive, recreational nature of the sample’s RG practice. Previous research has found that elite adolescent rhythmic gymnasts competing at an international and national level have expressed significant dissatisfaction with their own body image [32]. Similarly, concerns related to perceived body image have also been observed among female gymnasts at sub-national competition levels [33]. Additionally, a study by [34] observed that the mean ideal figure chosen by young competitive RG girls was significantly thinner than that selected by non-competitive ones, whereas no differences were detected between non-competitive RG girls and a control group.

Another noteworthy finding is that the BMI index levels observed in both the RG and control groups did not indicate a high prevalence of either obese/overweight girls [35]. Additionally, underweight participants were almost non-existent. This is consistent with the fact that somatic constitution is a key basic selection criteria in RG as athletes are generally slim and lean, with a prevalence of the ectomorphic component [36], resulting in low BMI values [37]. It could be argued that since body dissatisfaction was not associated with RG practice in our sample, BMI values were also unaffected. However, it should be noted that the girls with higher BMI values showed higher body dissatisfaction, which confirms the well-established direct association between these parameters [29].

Identifying and addressing body dissatisfaction is paramount for the holistic development and well-being of young gymnasts, ensuring that their athletic careers are both successful and healthy. Our results revealed the presence of body dissatisfaction among girls practicing RG at a recreational level. These findings underscore the necessity for coaches to receive basic training in addressing body image concerns and preventing the development of associated issues such as eating disorders or anxiety. Coaches should also be equipped to promote a positive body image, emphasizing performance and skill development over appearance in young RG practitioners.

Furthermore, the presence of body dissatisfaction in the control group underscores the importance of educating parents about the early signs and strategies for mitigating negative body image perceptions. By involving both coaches and parents in addressing body dissatisfaction, a supportive environment that fosters the overall well-being of young gymnasts can be created.

This study has several limitations that warrant consideration when interpreting the results presented here. Firstly, the cross-sectional design of this research restricts our ability to establish causality between variables. Secondly, we did not incorporate data on various socioeconomic factors (such as media exposure, parental influence, and peer relationships), which could act as confounders given their potential impact on the development of body dissatisfaction. Lastly, we encountered difficulty in matching controls to gymnasts based on factors beyond race, age, and BMI, introducing potential sources of bias in our comparisons.

## 5. Conclusions

Our findings indicate low levels of body dissatisfaction among young girls engaged in recreational RG, which are comparable to those observed in non-practicing peers. These results suggest that body dissatisfaction manifests early in life and may not be directly linked to participation in recreational RG. However, further studies involving more specific age groups are necessary to corroborate these findings.

## Figures and Tables

**Table 1:** Characteristics of the sample and body dissatisfaction outcomes.

	Rhythmic Group (n = 88)				Control Group (n = 88)				Total (n = 176)				
	Mean	SD	CI95%-L	CI95%-H	Mean	SD	CI95%-L	CI95%-H	Mean	SD	CI95%-L	CI95%-H	*p*
Age (years)	8.51	1.52	8.19	8.83	8.51	1.52	8.19	8.83	8.51	1.51	8.29	8.74	1.000
Weight (kg)	31.53	8.51	29.73	33.33	33.30	9.23	31.34	35.26	32.41	8.90	31.09	33.74	0.139
Heigth (m)	1.34	0.11	1.31	1.36	1.35	0.09	1.33	1.37	1.34	0.10	1.33	1.36	0.151
BMI (kg*m^−2^)	17.35	2.54	16.81	17.89	17.96	3.42	17.23	18.68	17.65	3.02	17.20	18.10	0.263
	n	%			n	%			n	%			
BMI categories													0.155
Underweight	1	1.14			5	5.68			6	3.41			
Normal weight	66	75.00			55	62.50			121	68.75			
Overweight	17	19.32			20	22.73			37	21.02			
Obese	4	4.55			8	9.09			12	6.82			
	**Mean**	**SD**			**Mean**	**SD**			**Mean**	**SD**			
Which picture looks the most like you?	3.96	0.89	3.77	4.15	4.06	1.08	3.83	4.29	4.01	0.99	3.86	4.16	0.341
Which picture shows the way you want to look?	3.02	1.01	2.80	3.23	2.99	1.17	2.75	3.24	3.01	1.09	2.84	3.17	0.984
Which picture shows the way you would like others see you?	3.13	1.05	2.91	3.35	3.15	1.26	2.88	3.41	3.14	1.16	2.97	3.31	0.742
Body dissatisfaction score	0.94	1.18	0.69	1.19	1.06	1.29	0.79	1.34	1.00	1.24	0.82	1.19	0.540

**Table 2:** Comparison of body dissatisfaction between gymnasts and controls.

	Rhythmic Group	Control Group	Total	*p*
Desire to be heavier	6 (6.8)	9 (10.2)	15 (8.5)	0.482
Satisfied with body image	26 (29.5)	20 (22.7)	46 (26.1)	
Desire to be thinner	56 (63.6)	59 (67.0)	115 (65.3)	

*p* values were calculated with the Chi-Squared test (categorical variables). A single *p* value is provided for every variable, regardless of the number of categories included.

**Table 3:** Body dissatisfaction according to body mass index strata.

Body Mass Index	Desire to Be Heavier	Satisfied with Body Image	Desire to Be Thinner	*p*
Underweight	0 (0)	3 (50.0)	3 (50.0)	0.006
Normal weight	15 (12.4)	37 (30.6)	69 (57.0)	
Overweight	0 (0)	5 (13.5)	32 (86.5)	
Obese	0 (0)	1 (8.3)	11 (91.7)	

*p* values were calculated with Chi-Square test (categorical variables). A single *p* value is provided for every variable, regardless of the number of categories included.

## Data Availability

The data presented in this study are available on request from the corresponding author due to ethical reasons.
